# Urgent Pericardiocentesis Is More Frequently Needed After Left Circumflex Coronary Artery Perforation

**DOI:** 10.3390/jcm9093043

**Published:** 2020-09-21

**Authors:** Michał A. Surdacki, Marcin Major, Michał Chyrchel, Paweł Kleczyński, Tomasz Rakowski, Leszek Bryniarski, Marek Ujda, Renata Wysocka, Witold Żmuda, Andrzej Wiśniewski, Marcin Nosal, Maciej Maliszewski, Marcin Rzeszutko, Jacek Legutko, Andrzej Surdacki, Stanisław Bartuś, Łukasz Rzeszutko

**Affiliations:** 1 Students’ Scientific Group at the Second Department of Cardiology, Jagiellonian University Medical College, 30-688 Cracow, Poland; msurdacki1997@gmail.com (M.A.S.); marcin.major@student.uj.edu.pl (M.M.); 2 Second Department of Cardiology, Institute of Cardiology, Jagiellonian University Medical College, 30-688 Cracow, Poland; michal.chyrchel@uj.edu.pl (M.C.); mcrakows@cyf-kr.edu.pl (T.R.); l_bryniarski@poczta.fm (L.B.); surdacki.andreas@gmx.net (A.S.); mbbartus@cyfronet.pl (S.B.); 3 Department of Cardiology and Cardiovascular Interventions, University Hospital, 30-688 Cracow, Poland; 4 Laboratory of Hemodynamics and Invasive Cardiology, District Hospital, 97-500 Radomsko, Poland; 5 Department of Interventional Cardiology, Institute of Cardiology, Jagiellonian University Medical College, 31-202 Cracow, Poland; kleczu@interia.pl (P.K.); jacek.legutko@uj.edu.pl (J.L.); 6 Subcarpathian Cardiovascular Intervention Center, 38-500 Sanok, Poland; wisniewskiandrzej@yahoo.com; 7 Center for Invasive Cardiology, Electrotherapy and Angiology, 38-400 Krosno, Poland; m.nosal@intercard.net.pl; 8 Interventional Cardiology Department, District Hospital, 37-450 Stalowa Wola, Poland; mujda@poczta.onet.pl; 9 Center for Invasive Cardiology, Electrotherapy and Angiology, 33-300 Nowy Sącz, Poland; r.korpak-wysocka@intercard.net.pl; 10 Center for Invasive Cardiology, Electrotherapy and Angiology, 32-600 Oświęcim, Poland; witowz@gmail.com; 11 Invasive Cardiology, Electrotherapy and Angiology Center, 27-400 Ostrowiec Świętokrzyski, Poland; mmaliszewski@gvmcarint.eu; 12 Department of Cardiology, District Hospital, 42-300 Myszków, Poland; marcin.rzeszutko@uj.edu.pl; 13 Department of Angiology, Jagiellonian University Medical College, 30-688 Cracow, Poland

**Keywords:** coronary perforation, cardiac tamponade, percutaneous coronary intervention, left circumflex coronary artery

## Abstract

**Background:** Coronary artery perforation (CAP) is a rare but potentially life-threatening complication of percutaneous coronary interventions (PCIs) due to the risk of cardiac tamponade. Strikingly, in contrast to numerous analyses of CAP predictors, only few studies were focused on the predictors of tamponade after PCI, once iatrogenic CAP has occurred. Our aim was to search for clinical and periprocedural characteristics, including the coronary artery involved, associated with the development of acute cardiac tamponade among patients experiencing CAP. **Methods:** From the medical records of nine centers of invasive cardiology in southern Poland, we retrospectively selected 81 patients (80% with acute myocardial infarction) who had iatrogenic CAP with a visible extravasation jet during angiography (corresponding to type III CAP by the Ellis classification, CAP_III_) over a 15-year period (2005–2019). Clinical, angiographic and periprocedural characteristics were compared between the patients who developed acute cardiac tamponade requiring urgent pericardiocentesis in the cathlab (n = 21) and those with CAP_III_ and without tamponade (n = 60). **Results:** CAP_III_ were situated in the left anterior descending artery (LAD) or its diagonal branches (51%, n = 41), right coronary artery (RCA) (24%, n = 19), left circumflex coronary artery (LCx) (16%, n = 13), its obtuse marginal branches (7%, n = 6) and left main coronary artery (2%, n = 2). Acute cardiac tamponade occurred in 24% (10 of 41), 21% (4 of 19) and 37% (7 of 19) patients who experienced CAP_III_ in the territory of LAD, RCA and LCx, respectively. There were no significant differences in the need for urgent pericardiocentesis (37%) in patients with CAP_III_ in LCx territory (i.e., the LCx or its obtuse marginal branches) compared to CAP_III_ in the remaining coronary arteries (23%) (*p* = 0.24). However, when CAP_III_ in the LCx were separated from CAP_III_ in obtuse marginal branches, urgent pericardiocentesis was more frequently performed in patients with CAP_III_ in the LCx (54%, 7 of 13) compared to subjects with CAP_III_ in an artery other than the LCx (21%, 14 of 68) (*p* = 0.03). The direction of this tendency remained consistent regardless of CAP management: prolonged balloon inflation only (n = 26, 67% vs. 13%, *p* = 0.08) or balloon inflation with subsequent stent implantation (n = 55, 50% vs. 24%, *p* = 0.13). Besides LCx involvement, no significant differences in other characteristics were observed between patients according to the need of urgent pericardiocentesis. **Conclusions:** CAP_III_ in the LCx appears to lead to a higher risk of acute cardiac tamponade compared to perforations involving other coronary arteries. This association may possibly be linked to distinct features of LCx anatomy and/or well-recognized delays in diagnosis and management of LCx-related acute coronary syndromes.

## 1. Introduction

Coronary artery perforation (CAP) is a rare but potentially dangerous complication of percutaneous coronary interventions (PCIs) due to the risk of cardiac tamponade, highest in grade III CAP (CAP_III_) according to the classical Ellis criteria [1,2,3,4,5,6]. Strikingly, in contrast to numerous analyses of CAP predictors [4,7,8,9,10,11], only few studies were focused on the predictors of tamponade after PCI, once iatrogenic CAP has occurred [12,13,14].

There are partially inconsistent reports regarding the coronary artery spatial distribution of CAP, considered as a proportion of the treated PCI target vessels [4,8,9,10,11]. Our aim was to search for clinical and periprocedural characteristics, including the coronary artery involved, associated with the development of acute tamponade among patients experiencing CAP_III_.

We hypothesized that perforations in some specific coronary artery segments may lead to a higher risk of acute tamponade.

## 2. Materials and Methods

### 2.1. Protocol

From the medical records of nine centers of invasive cardiology in southern Poland, we retrospectively selected 81 patients (80% with acute myocardial infarction) who experienced CAP grade III by the Ellis criteria (CAP_III_, i.e., with visible contrast extravasation through a frank perforation, diagnosed in the cathlab [1]) over a 15-year period (2005–2019). Patients with CAP_III_ during an attempted angioplasty of chronic coronary occlusion or those caused by a guidewire tip were a priori excluded from the analysis. On the basis of an approximate total number of coronary interventions performed during that period, the incidence of CAP_III_ could be estimated to be between 0.09% and 0.13%.

Acute cardiac tamponade was diagnosed in the cathlab patients with rapidly developing symptoms (systemic hypotension, pulsus paradoxus, tachycardia and raised jugular venous pressure) with pericardial fluid collection and echocardiographic signs of tamponade, including diastolic collapse of the right cardiac chambers, abnormal interventricular septum motion, exaggerated respiratory variation of transvalvular flow velocities and dilated inferior vena cava.

Clinical, angiographic and periprocedural characteristics were compared between the patients with CAP_III_ who developed acute cardiac tamponade requiring urgent pericardiocentesis in the cathlab (n = 21) and those with CAP_III_ without acute tamponade (n = 60).

The study protocol was approved by the ethical committee of our university, including a waiver of patients’ informed consent to data analysis owing to a retrospective study design (Approval No. 1072.6120.85.2020 of 23 April 2020).

### 2.2. Statistical Analysis

Data are shown as means and standard deviation or numbers and percentages. Intergroup comparisons were performed by a two-tailed Student’s *t*-test or Fisher’s exact test for continuous and dichotomous characteristics, respectively. The concordance with a Gaussian distribution and the homogeneity of variance were checked by the Lilliefors test and Levene’s test, respectively. In order to identify independent predictors of acute tamponade, multivariate logistic regression analysis was done, including the variables with a univariate intergroup *p*-value below 0.20 as potential covariates. The odds ratio (OR) of developing acute tamponade with 95% confidence intervals (CIs) and respective *p*-values were presented. The goodness-of-fit of the regression model was estimated by the Hosmer–Lemeshow test. A *p*-value below 0.05 was inferred to be significant.

## 3. Results

Most CAP_III_ were situated in the left anterior descending artery (LAD) or its diagonal branches (51%, n = 41), followed by the right coronary artery (RCA) (24%, n = 19), left circumflex coronary artery (LCx) (16%, n = 13), its obtuse marginal branches (7%, n = 6) and left main coronary artery (2%, n = 2).

Acute cardiac tamponade requiring urgent pericardiocentesis in the cathlab occurred in 24% (10 of 41), 21% (4 of 19) and 37% (7 of 19) patients who experienced CAP_III_ in the territory of the LAD, RCA and LCx, respectively.

**Figure 1:**
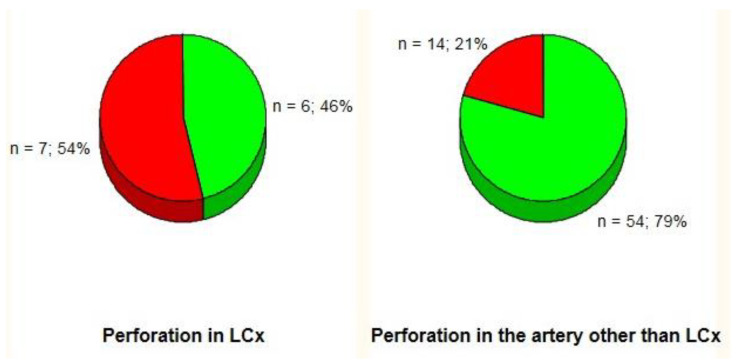
Incidence of acute cardiac tamponade (red area) by perforation site. LCx: left circumflex coronary artery.

**Figure 2:**
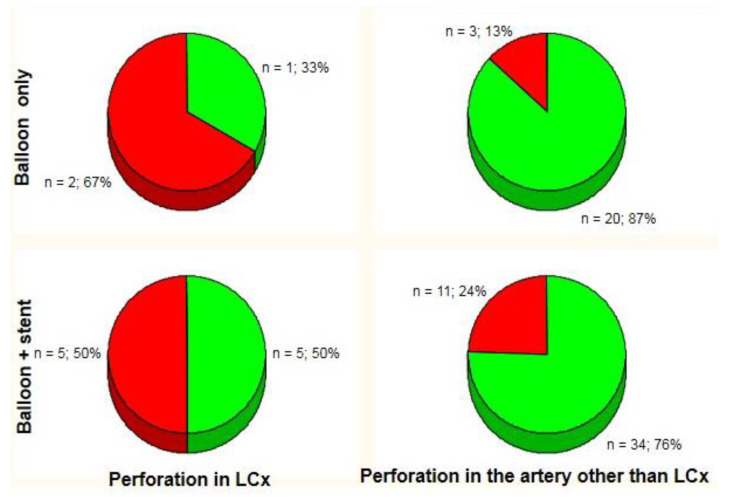
Incidence of acute cardiac tamponade (red area) by management strategy and perforation site. LCx: left circumflex coronary artery.

There were no significant differences in the need for urgent pericardiocentesis (37%, 7 of 19) in patients with CAP_III_ in LCx territory (i.e., the LCx and its obtuse marginal branches) compared to CAP_III_ in the remaining coronary arteries (23%, 14 of 62) (*p* = 0.24). Obtuse marginal branches of the LCx were defined as its side branches running in general to the area of obtuse margin of the heart [15]. However, when CAP_III_ in the LCx (situated in the proximal or middle segments in all subjects) were separated from CAP_III_ in obtuse marginal branches, urgent pericardiocentesis was more frequently performed in patients with CAP_III_ in the LCx (54%, 7 of 13) compared to subjects with CAP_III_ in an artery other than the LCx (21%, 14 of 68) (*p* = 0.03) (Figure 1). The direction of this tendency remained consistent regardless of CAP management: prolonged balloon inflation (PBI) only (n = 26, 67% vs. 13%, *p* = 0.08) or PBI with subsequent implantation of a covered or standard stent (n = 55, 50% vs. 24%, *p* = 0.13) (Figure 2).

Besides LCx involvement, no significant differences in other characteristics were observed between patients according to the need of urgent pericardiocentesis (Table 1).

By multivariate logistic regression (*p* = 0.76 by the goodness-of-fit Hosmer–Lemeshow test), the association between CAP_III_ in the LCx and the risk of developing acute tamponade retained statistical significance (OR: 4.3 (95% CI, 1.2–15.5) for CAP_III_ in the LCx vs. non-LCx, *p* = 0.02). Additionally, a weak tendency towards a higher risk of tamponade was observed in women (OR: 2.5 (0.9–7.3), *p* = 0.09).

## 4. Discussion

Our salient finding was a higher incidence of acute cardiac tamponade requiring pericardiocentesis after LCx perforation compared to CAP_III_ in other coronary arteries. To the best of our knowledge, our report is one of the several largest studies which have been published on CAP so far, especially grade III CAP.

This observation supplements earlier reports of distinct features of acute coronary syndromes with the culprit lesion in the LCx. These differences include a lower sensitivity of ECG to detect postero-lateral myocardial ischemia and a more frequent presentation as non-ST segment elevation myocardial infarction (NSTEMI) compared to other culprit vessels with consequent delay in PCI.

### 4.1. Comparison with Previous Studies

#### 4.1.1. Incidence of Cardiac Tamponade after Coronary Perforation

In our study, acute cardiac tamponade requiring pericardiocentesis was observed in 21 out of 81 CAP_III_ (26%), which is comparable to the respective percentages (29% (16 out of 56) and 36% (26 out of 73)) in some recent reports on CAP [14,16], but lower than in earlier, smaller series, ranging from 37.5% to 67% of CAP_III_ [5,17]. In a recent large registry-based report that differentiated between acute and late tamponade [18], acute cardiac tamponade (i.e., diagnosed in the cathlab) occurred in 153 of 1008 CAP (15%). Nevertheless, the study subjects were not characterized according to the Ellis criteria [18]. Similarly, the CAP type was not shown in the reports by Guttmann et al. [10] (29% of 149 CAP, with the majority (79%) manifesting as acute tamponade) and Kinnaird et al. [9] (14% of 1762 CAP).

#### 4.1.2. Incidence of Iatrogenic Cardiac Tamponade According to the Vessel Involved

Stathopoulos et al. [14], who analyzed a total of 73 patients experiencing CAP_III_, observed an insignificantly higher proportion of cardiac tamponade after CAP_III_ in the LAD (58% and 36% for CAP_III_ with and without tamponade, respectively) as opposite to the RCA (15% and 32%), while the respective proportions were similar for CAP_III_ involving the LCx (27% and 23%). This pattern was similar to those recently shown by Harnek et al. [18] for acute tamponade. Nevertheless, the Ellis grade was not reported in that registry encompassing 243,149 patients and 1008 CAP episodes [18].

Fejka et al. [13] described an insignificantly lower proportion of the LAD in 31 patients who developed tamponade (26%) compared to 25,697 remaining PCI subjects free of this complication, in whom the LAD was the intervention site (40%), whereas the percentages were similar for the RCA (39% vs. 34%) and the LCx (23% vs. 28%). However, they did not compare patients with post-PCI cardiac tamponade with their counterparts with CAP without tamponade, as in the present study. In an early report by Von Sohsten et al. [12], cardiac tamponade within 36 h after PCI resulted from right ventricular perforation by a temporary pacing wire in 7 out of 15 patients with CAP, which limits comparisons with other studies.

#### 4.1.3. Coronary Artery Spatial Distribution of Coronary Perforations

In the present study, most CAP_III_ involved LAD territory (51%), followed by RCA (24%) and LCx (23%) territories, which is consistent with the range of respective proportions in the previously reported series of CAP (LAD: 25–52%; RCA: 23–50%; LCx: 18–29%) [4,6,8,9,10,14].

In contrast to the vast majority of studies which encompassed CAP grades I–III, only one analysis of a large dataset, including 24,465 patients [16], was precisely focused on the predictors of grade III CAP, complicating 0.23% of PCI. Additionally, in a report by Rakowski et al. [11], who analyzed 344,517 coronary interventions, the vast majority of CAP, diagnosed in the cathlab and occurring in 0.17% of patients, presumably corresponded to CAP_III_. In these two studies [11,16], CAP predictors, differentiating those with versus without CAP, included age, female sex, previous CABG, multivessel CAD, PCI of complex lesions or chronic total occlusions, and use of rotablative devices or intravascular ultrasound. In addition, in one of the aforementioned reports [11], the risk of CAP was lower for PCI of a lesion in the proximal or medium part of the LCx, being higher for the RCA and mid-LAD. Nevertheless, in that study [11] the intervention site was not identified as an independent CAP predictor by multivariate logistic regression, which is consistent with an earlier report by Al-Lamee et al. [16], limited to CAP_III_ perforations.

Irrespective of these rather minor inconsistencies, the distribution of culprit lesions in our study group appears comparable to that in the majority of the previously cited studies, with most CAP involving the LAD, followed by the RCA and then the LCx [6,9,10,14,16].

### 4.2. Mechanistic Considerations – Clinical Implications of LCx Involvement as a Culprit Vessel in Acute Coronary Syndromes

The mechanism of our observation is unknown and we can only speculate on this issue. Hypothetically, the well-recognized delays in the diagnosis and management of LCx-related acute myocardial infarction [19,20] could contribute to an increased risk of cardiac tamponade after CAP, possibly through the time-dependent development of structural changes in the involved arterial wall before the delayed intervention on the LCx. In agreement with this concept, in 1500 patients with an acute myocardial infarction due to acute coronary occlusion, the percentage of patients who underwent PCI more than 24 h from symptom onset was higher for the LCx (30%) than the LAD (17%) or RCA (20%) [19], which was explained by a lower sensitivity of ST segment elevation in both standard and extended precordial ECG leads in the detection of acute LCx occlusion [20]. This hypothesis is also consistent with an underrepresentation of the LCx as the culprit artery among patients with ST segment elevation myocardial infarction (STEMI) [21,22,23]. On the other hand, among our patients with an LCx-related acute coronary syndrome and CAP_III_, the percentage of STEMI was higher in the subjects who later developed cardiac tamponade compared to those free of this complication (40% vs. 25%), which contradicts the above mechanism.

Nevertheless, alternative explanations of the observed relations can also be proposed, linked to specific features of LCx anatomy and biomechanical factors. Ghanim et al. [23] reported an about six-fold lower systolic coronary shortening along the artery’s long axis (i.e., coronary longitudinal strain) in the proximal and middle LCx (1.5%) compared to the LAD or RCA (about 9–10%). Mechanistically, the proximal and middle segments of the LCx run along the circumferential axis of the LV base in the atrioventricular groove, whereas the LAD, most of the RCA, the distal LCx and obtuse marginal branches travel approximately along the LV longitudinal axis, consistent with the direction of LV systolic shortening [23]. Notably, in a large registry, Rakowski et al. [11] reported a lower risk of CAP in LCx territory, which was largely mediated by a decreased incidence of CAP in the proximal-to-mid LCx, but not the distal Cx or its obtuse marginal branches.

Accordingly, the lower chronic longitudinal strain in the proximal and middle LCx would actually appear to be a protective factor against severe CAP in this territory. We can only cautiously hypothesize that unknown and possibly related mechanisms might also counterintuitively predispose to acute post-CAP tamponade in that intervention site. Nevertheless, further studies are warranted to validate our preliminary findings and their hypothetical mechanistic interpretations.

### 4.3. Study Limitations

First, beyond a low statistical power of our study and the consequent limited validity of any cause-and-effect considerations, our retrospective analysis was based exclusively on available cathlab medical records. Therefore, episodes of late tamponade [10,18]—occurring in the ward, not in the cathlab—were not included in the analysis. Second, for the same reason, clinical outcomes of the patients experiencing acute tamponade were not shown in the present study. Additionally, we had no access to the complete data on the necessity of cardiac surgery during the index hospitalization in the ward. Indeed, emergency cardiac surgery, albeit seldom performed as a primary management strategy of CAP, may be required to alleviate tamponade after unsuccessful percutaneous pericardiocentesis, and also as a bail-out coronary artery bypass grafting and surgical repair of coronary perforations [5,6,10,16,17]. Nevertheless, since our district invasive cardiology centers do not have on-site cardiac surgery, surgical management is extremely rare in this setting due to transport-related delays in the transfer of a patient with a life-threatening condition. Third, owing to a limited availability of medical records, we were also unable to provide detailed data on medication use prior to the index coronary intervention, except for oral anticoagulants. Nevertheless, to the best of our knowledge, our report belongs to the several largest published studies in this field.

## 5. Conclusions

Iatrogenic LCx perforation appears to lead to a higher risk of acute cardiac tamponade compared to perforations involving other coronary arteries. This association may possibly be linked to distinct features of LCx anatomy and/or well-recognized delays in diagnosis and management of LCx-related acute coronary syndromes.

## Figures and Tables

**Table 1:** Characteristics of patients experiencing grade III coronary artery perforation with and without acute cardiac tamponade requiring urgent pericardiocentesis in the cathlab.

Characteristic	Acute Tamponade n = 21	No Tamponade n = 60	*p*-Value ^a^
Baseline clinical characteristics			
Age (years)	70 ± 9	70 ± 10	0.98
Men/Women (%)	38/62	62/38	0.08
Body mass index (kg/m^2^)	25.8 ± 4.3	27.3 ± 4.6	0.22
Acute coronary syndrome/stable angina (%)	81/19	78/22	1
Hypertension (%)	81	73	0.57
Diabetes (%)	33	33	1
Estimated GFR (mL/min per 1.73 m^2^)	73 ± 21	70 ± 21	0.55
Overt heart failure (%)	5	5	1
Ejection fraction (%)	45 ± 17	42 ± 11	0.44
Oral anticoagulants before intervention (%)	24	12	0.28
Treated vessel (%)			
LAD and/or Dg_1/2_	48	52	0.80
RCA	19	25	0.77
LCx	33	10	**0.03**
Mg_1/2_	0	10	0.33
LMCA	0	3	1
Procedural data			
Lesion predilation (%)	76	80	0.76
Predilation balloon maximal pressure (atm.)	16.1 ± 4.6	13.5 ± 5.8	0.13
Predilation balloon size (mm)	2.9 ± 0.8	2.6 ± 0.6	0.15
Predilation balloon length (mm)	16.3 ± 3.2	17.6 ± 3.5	0.22
Stent deployment maximal pressure (atm.)	15.0 ± 2.9	14.6 ± 3.1	0.70
Maximal stent diameter (mm)	3.4 ± 0.7	3.3 ± 0.7	0.91
Total stent length (mm)	26.9 ± 14.4	22.8 ± 10.0	0.23
Stent postdilation (%)	29	45	0.21
Postdilation balloon maximal pressure (atm.)	19.3 ± 10.3	17.1 ± 6.1	0.48
Postdilation balloon size (mm)	3.4 ± 0.5	3.7 ± 0.9	0.47
Postdilation balloon length (mm)	12.2 ± 2.3	16.1 ± 6.4	0.21
Cutting balloon (%)	5	2	0.45

Data are shown as mean ± SD or %. ^a^ Significant intergroup differences are marked in bold. Dg_1/2_: first or second diagonal branch; GFR: glomerular filtration rate by the CKD-EPI formula; LAD: left anterior descending artery; LCx: left circumflex coronary artery; LMCA: left main coronary artery; Mg_1/2_: first or second obtuse marginal branch; n.s.: non-significant; RCA: right coronary artery.
